# Bayesian inference for nonlinear mixed-effects location scale and interval-censoring cure-survival models: An application to pregnancy miscarriage

**DOI:** 10.1177/09622802251345485

**Published:** 2025-05-29

**Authors:** Danilo Alvares, Cristian Meza, Rolando De la Cruz

**Affiliations:** 1 MRC Biostatistics Unit, 2152 University of Cambridge, Cambridge, UK; 2 INGEMAT-CIMFAV, Faculty of Engineering, 28068 Universidad de Valparaíso, Valparaiso, Chile; 3 Faculty of Engineering and Sciences, 28034 Universidad Adolfo Ibáñez, Penalolen, Chile; 4 Data Observatory Foundation, ANID Technology Center, Chile

**Keywords:** Joint models, longitudinal data, mixed-effects location scale, three-parameter logistic model, time-to-event

## Abstract

Motivated by a pregnancy miscarriage study, we propose a Bayesian joint model for longitudinal and time-to-event outcomes that takes into account different complexities of the problem. In particular, the longitudinal process is modeled by means of a nonlinear specification with subject-specific error variance. In addition, the exact time of fetal death is unknown, and a subgroup of women is not susceptible to miscarriage. Hence, we model the survival process via a mixture cure model for interval-censored data. Finally, both processes are linked through the subject-specific longitudinal mean and variance. A simulation study is conducted in order to validate our joint model. In the real application, we use individual weighted and Cox-Snell residuals to assess the goodness-of-fit of our proposal versus a joint model that shares only the subject-specific longitudinal mean (standard approach). In addition, the leave-one-out cross-validation criterion is applied to compare the predictive ability of both models.

## 1. Introduction

In Obstetrics research, a recurring interest is to study longitudinal beta-human chorionic gonadotropin ( β -HCG) hormone measurements from women during the first quarter of their pregnancies and the pregnancy outcome from some women who had complications leading to miscarriage.^1^ During the early stages of pregnancy, it is important to consider how the fluctuation in hormone concentration happens within such a framework, since it might alter the pregnancy’s outcome.

This problem was first modeled by Marshall and Barón,^2^ where they proposed a nonlinear mixed-effects model using a parametric logistic function to model hormone concentration over time using maximum likelihood estimates, and De la Cruz-Mesía and Quintana^3^ provided a Bayesian approach. To model this biomarker and a pregnancy outcome together, De la Cruz et al.^4,5^ explain the relationship between a binary response (pregnancy outcome) and the characteristics of longitudinal measurements (hormone levels). So, the joint model is made up of a logistic regression that has individual-specific random effects from a nonlinear mixed-effects model as variables. In De la Cruz et al.,^4^ the authors compared a number of estimation techniques, including the Laplacian approximation, the naïve two-stage method, best linear unbiased prediction, and Gaussian and adaptive Gaussian quadratures. In De la Cruz et al.,^5^ the authors proposed a Bayesian inference based on a Markov chain Monte Carlo sampler and introduced autocorrelated errors into the joint model.

Clinicians have a critical interest in being able to evaluate the relationship between longitudinally recorded β -HCG and time to early miscarriage. So, the use of subject-specific random effects from a mixed-effects model for longitudinal β -HCG data as predictors in a survival model is a typical joint modeling strategy proposed to accomplish this goal. However, it frequently occurs while examining time-to-event data that a portion of participants will never experience the relevant event. From a modeling perspective, the so-called cure models^6^ incorporate such a characteristic, in which participants are believed to have been cured, and these event periods are considered limitless. Hence, our proposal includes a mixture cure specification^7^ to model the time until fetal death, where such times are interval-censored. In addition, a nonlinear mixed-effects location scale (MELS) model^8^ is proposed to capture the trajectories of β -HCG hormone and share the within-subject variability.

The remainder of the paper is organized as follows. In Section 2, we present a Chilean pregnancy miscarriage dataset, which was the motivation for the modeling developed in this paper. In Section 3, we gradually introduce the proposed model formulation, as well as its likelihood function and prior distributions. In Section 4, we evaluate the performance of our proposal with a simulation study. In Section 5, we discuss the results of two specifications of shared elements between the longitudinal and survival submodels. Finally, in Section 6, we conclude with a few general remarks. The models implemented in this paper were written in Stan^9^ and are available at www.github.com/daniloalvares/BJM-MELS-Cure.

## 2. Pregnancy miscarriage data

Our motivation comes from a clinical trial study in a Chilean private assisted reproduction center. The data consist of longitudinal β -HCG hormone measurements (in a log_10_ scale, which from now on will be denoted as log( β -HCG)) from 173 young women during the first quarter of their pregnancies. This hormone is produced by the placenta during pregnancy. Typically, β -HCG levels increase steadily until the end of the first trimester (10 weeks of pregnancy), then decline as the pregnancy progresses.^10^

**Figure 1.**
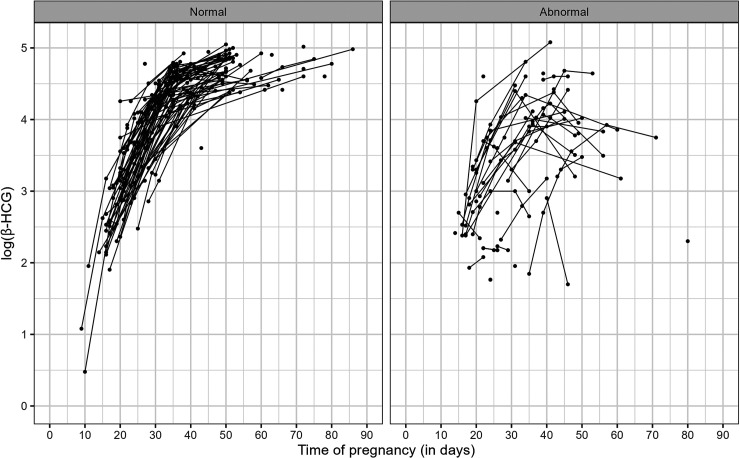
Longitudinal measurements of log( β -HCG) by pregnancy group. β -HCG: beta human chorionic gonadotropin.

In our study data, 49 women had complications that led to a miscarriage. From now on, the term *abnormal* will be used to refer to this women’s group. In contrast, 124 women had regular pregnancies and formed the *normal* group. Unfortunately, β -HCG levels during the first weeks of pregnancy are recorded infrequently and not always at the same stage of pregnancy for every woman.^11,12^ Figure 1 shows the β -HCG profiles over time for both groups.

Figure 1 reveals that there is a notable difference between the longitudinal profiles of each group. In particular, the normal group has an increasing, nonlinear, and homogeneous evolution, while the abnormal group trajectories do not follow a clear pattern, but they have lower β -HCG hormone levels than the normal group and much more variability. These preliminary visual analyzes suggest that the distribution of longitudinal measurements depends on the pregnancy group. Hence, we can assume that the shared characteristics of β -HCG trajectories potentially help to explain the time until miscarriage. Furthermore, as previously pointed out, we can also see that both groups have irregular frequency and number of measurements. Table 1 shows the frequency of longitudinal measurements by pregnancy group.

**Table 1.** Frequency of the number of individual longitudinal measurements by pregnancy group.

Pregnancy group	Number of longitudinal measurements					
	1	2	3	4	5	6
Normal	35	44	42	3	0	0
Abnormal	17	9	16	5	1	1

**Figure 2.**
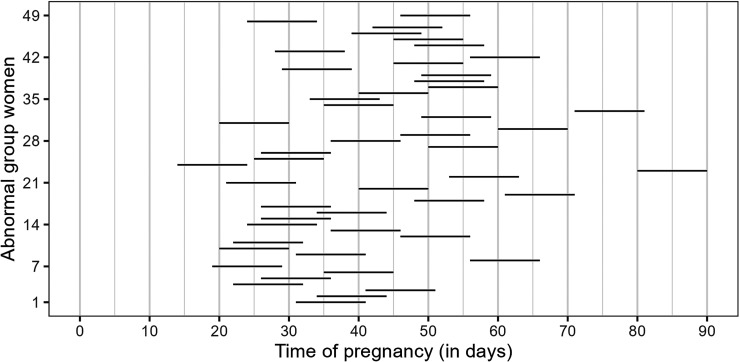
Time interval (10-day range from the last measurement of the β -HCG hormone) in which fetal death occurred for abnormal group women. β -HCG: beta human chorionic gonadotropin.

The main objective of this study is to analyze the association of the β -HCG levels with the time until fetal death. Here, it is important to note that only the abnormal group experiences the event of interest (fetal death). Another relevant characteristic of the problem is that the exact time of fetal death is unknown. However, miscarriage symptoms and emergency medical follow-up usually occur within a period of 10 days (regular time for miscarriage detection through medical visit and/or clinical examination in Chile) after the last measurement of the β -HCG hormone.^13^ Assuming such a time window, Figure 2 shows the time interval in which fetal death occurred for the 49 women in the abnormal group. We can observe that most women had an early pregnancy loss between the 20th and 60th day of pregnancy.

The data of this study present a severe limitation which is the absence of (baseline) covariates. So, conclusions are limited to the knowledge that can be extracted from longitudinal measurements and survival times.

## 3. The Bayesian joint model

Conceptually, a joint model connects two or more processes through shared terms.^14,15^ Here, such processes are described by a longitudinal submodel for the β -HCG hormone (endogenous time-varying covariate) and a survival submodel for the time until fetal death. Each element of our joint modeling proposal is introduced in the following.

Let $y_{i}(t)$ be the log( β -HCG) measurement associated with the i th woman, i=1,…,N , measured at time t . It is worth noting that log( β -HCG) is always positive once β -HCG for pregnant women is greater than 1. Define the conditional distribution of $y_{i}(t)$ given θ (parameters), $b_{i}$ (random effects), and σ (error standard deviation) as a generic additive error model:

(1) $$y_{i}(t|\theta ,b_{i},\sigma )=\mu_{i}(t|\theta ,b_{i})+\epsilon_{i}(t|\sigma )$$

where $\mu_{i}(t|\theta ,b_{i})$ represents the mean response at time t and $\epsilon_{i}(t|\sigma )$ is a residual error. We assume that random effects, $b_{i}$ , given Σ , follow a multivariate normal distribution with zero-mean vector and variance-covariance matrix Σ . The residual errors are assumed to be conditionally independent and identically distributed as $\epsilon_{i}(t|\sigma )∼\text{Normal}(0,\sigma^{2})$ .

The survival submodel aims to model the time until fetal death, which occurred within 10 days after the last measurement of the β -HCG hormone. Here, two important characteristics should be noted: (i) A part of the women (normal group) are not susceptible to the event of interest, and (ii) the exact time of fetal death is unknown, leading to interval-censored observations.

From a modeling perspective, (i) requires a mixture cure model, since some women have given birth and are therefore not susceptible to fetal loss.^7^ Specifically, let Z be a binary random variable defined as 0 for a susceptible woman and 1 for an immune woman. So, the *incidence model* is given by $\text{P}(Z_{i}=1)=\eta =1/(1+\exp\;(-\nu ))$ , where η represents the cure fraction. In addition, let $T_{i}$ be the time until fetal death for the susceptible woman i (i.e. $T_{i}$ conditional on $Z_{i}=0$ ), so the *latency model* is expressed through a proportional hazard specification:

(2) $$h(t|\phi ,\lambda ,\alpha_{1},\theta ,b_{i})=\phi \;t^{\phi -1}\exp\;\left\{\lambda +\alpha_{1}\mu_{i}(t|\theta ,b_{i})\right\}$$

where ϕ and λ are Weibull hazard shape and log-scale parameters, respectively. The term $\mu_{i}(t|\theta ,b_{i})$ has the role of connecting the longitudinal and survival submodels, while $\alpha_{1}$ measures the strength of this association. We chose a Weibull baseline hazard because a similar study^13^ using the same data corroborated that this specification is sufficiently adequate, but other alternatives could also be employed, such as piecewise and spline functions.^16^

### 3.1. Adding the three-parameter logistic specification

Typically, the longitudinal submodel (1) of a joint model is defined as a linear mixed-effects model with random intercept and slope.^17–22^ However, β -HCG hormone trajectories clearly show nonlinear patterns (see Figure 1) that are not captured well with such a structure. To get around this issue, Marshall and Barón^2^ successfully proposed a three-parameter logistic specification given by:

(3) $$\mu_{i}(t|\theta ,b_{i})=\frac{a_{i1}}{1+\exp\;\left\{-\frac{\left(t-a_{i2}\right)}{a_{i3}}\right\}}$$

where $a_{i1}=\exp\;\{\theta_{1}+b_{i1}\}$ , $a_{i2}=\exp\;\{\theta_{2}+b_{i2}\}$ , and $a_{i3}=\exp\;\{\theta_{3}+b_{i3}\}$ . The joint model (1)-(2) using the three-parameter logistic specification (3) will be called the *reference joint model*.

### 3.2. Adding the MELS specification

Figure 1 suggests that the variability of longitudinal trajectories may be a risk factor for the time until fetal death. In order to incorporate this characteristic into the modeling, we specify within-subject variances using a MELS model^8^ as follows:

(4) $$y_{i}(t|\theta ,b_{i})=\mu_{i}(t|\theta ,b_{i})+\epsilon_{i}(t|\sigma_{i})$$

where $\sigma_{i}=\exp\;\{\theta_{4}\}$ for $n_{i}=1$ (number of longitudinal measurements) and $\sigma_{i}=\exp\;\{\theta_{4}+b_{i4}\}$ for $n_{i}\geq 2$ . Hence, following the proposal of Barrett et al.,^23^ we rewrite the hazard function (2) including $\sigma_{i}^{2}$ as a second shared term:

(5) $$h(t|\phi ,\lambda ,\alpha_{1},\alpha_{2},\theta ,b_{i})=\phi \;t^{\phi -1}\exp\;\left\{\lambda +\alpha_{1}\mu_{i}(t|\theta ,b_{i})+\alpha_{2}\sigma_{i}^{2}\right\}$$

### 3.3. Likelihood and priors

The likelihood function of the full parameter vector and random effects of the joint model (4)-(5) using the three-parameter logistic specification (3) is given by:

(6) $$L(\Phi )=\prod_{i=1}^{N}\prod_{j=1}^{n_{i}}f(y_{ij}|\Phi )f(b_{i}|\Sigma )\prod_{i\in I}(1-\eta )[S(t_{i,L}|\Phi )-S(t_{i,R}|\Phi )]\prod_{i\in R}[\eta +(1-\eta )S(t_{i}|\Phi )]$$

where $y_{ij}$ is the value of log( β -HCG) for the i th woman at visit j ; ( $t_{i,L},t_{i,R}$ ) is the time interval in which the miscarriage occurred for a woman i belonging to the abnormal group ( I ); $t_{i}$ is the right-censored observation (last longitudinal measurement time) of a woman i belonging to the normal group ( R ); $\Phi =(\theta ,\nu ,\phi ,\lambda ,\alpha_{1},\alpha_{2},\Sigma ,b_{1},\ldots ,b_{n})$ denotes the full parameter vector and random effects; $f(y_{ij}|\Phi )$ represents the conditional probability density function of $y_{ij}$ given Φ described in (4) with respective random effects density function denoted by $f(b_{i}|\Sigma )$ ; and S(t∣Φ) is the survival function derived from (5).

We assume independent and proper prior distributions.^24^ More specifically, all longitudinal and survival fixed effects, $\left(\theta_{1},\theta_{2},\theta_{3},\theta_{4},\nu ,\lambda ,\alpha_{1},\alpha_{2}\right)$ , follow a Normal( $0,10^{2}$ ); the error variance, $\sigma^{2}$ , and the Weibull shape parameter, ϕ , follow a half-Cauchy( 0,1 );^25^ and the random effects variance-covariance matrix Σ follows an inverse-Wishart( $I_{5},4$ ),^26^ where $I_{5}$ represents a 5×5 identity matrix. We previously investigated the sensitivity of our prior distributions compared to vaguer ones, Normal( $0,100^{2}$ ) and half-Cauchy( 0,10 ), and we concluded that our choice is weakly informative, since the results were equivalent, differing only in computational time.

## 4. Simulation study

We conducted a simulation study to evaluate the performance of our proposal in estimating the parameters θ and α , compared to the reference joint model. We explored two scenarios: (I) simulated data from the joint model (1)-(2) (share $\mu_{i}$ ) and (II) simulated data from the joint model (4)-(5) (share $\mu_{i}$ and $\sigma_{i}^{2}$ ). In both cases, we considered 1–4 and 5–10 longitudinal measurements per individual, N=200 (sample size), and 1000 repetitions. The specification of the parameters is based on the fit of each model with the pregnancy miscarriage data (see Section 2). Specifically, Scenario I: $\theta_{1}=1.5$ , $\theta_{2}=2.7$ , $\theta_{3}=1.9$ , σ=0.25 , λ=−14.5 , ϕ=4 , $\alpha_{1}=-0.6$ , and Σ=diag(0.02,0.08,0.17) ; Scenario II: $\theta_{1}=1.5$ , $\theta_{2}=2.7$ , $\theta_{3}=1.9$ , $\theta_{4}=-2$ , λ=−14.7 , ϕ=3.9 , $\alpha_{1}=-0.4$ , $\alpha_{2}=3.1$ , and Σ=diag(0.02,0.07,0.1,0.6) . Table 2 summarizes the results and terms of bias and 95% coverage probability.

**Table 2.** Bias and 95% CP for θ and α in Scenarios I (true model: (1)-(2)) and II (true model: (4)-(5)) considering 1–4 and 5–10 LMPI with N=200 .

		1–4 LMPI			5–10 LMPI		
		Joint model (1)-(2)		Joint model (4)-(5)		Joint model (1)-(2)		Joint model (4)-(5)	
Scenario	Parameter	Bias	95% CP	Bias	95% CP	Bias	95% CP	Bias	95% CP
	$\theta_{1}$	− 0.007	0.98	− 0.008	0.97	− 0.005	0.95	− 0.003	0.95
	$\theta_{2}$	− 0.020	0.97	− 0.035	0.98	− 0.015	0.94	− 0.026	0.94
	$\theta_{3}$	− 0.011	0.98	− 0.001	0.96	− 0.002	0.94	− 0.001	0.95
I	$\alpha_{1}$	0.046	0.92	0.032	0.95	0.002	0.96	− 0.008	0.96
	$\theta_{1}$	− 0.005	0.98	− 0.003	0.96	− 0.003	0.96	− 0.002	0.95
	$\theta_{2}$	− 0.018	0.94	− 0.014	0.94	− 0.011	0.95	− 0.008	0.94
	$\theta_{3}$	− 0.010	0.98	− 0.008	0.97	− 0.008	0.96	− 0.005	0.96
	$\theta_{4}$	–	–	0.032	0.93	–	–	0.004	0.95
	$\alpha_{1}$	− 0.331	0.83	− 0.021	0.93	− 0.210	0.89	− 0.012	0.94
II	$\alpha_{2}$	–	–	-0.024	0.93	–	–	0.013	0.95

CP: coverage probability; LMPI: longitudinal measurements per individual.

The population parameters θ ’s are well estimated in all scenarios for both joint models. In Scenario I, where (1)-(2) is the true model, our proposal appropriately estimates the association parameter $\alpha_{1}$ even with few longitudinal measurements per individual. In Scenario II, where (4)-(5) is the true model, our proposal is also suitable, as expected, while the reference joint model produces biased estimates of the parameter associated with the shared mean response.

## 5. Application

We implemented Bayesian joint models (1)-(2) (share $\mu_{i}$ ) and (4)-(5) (share $\mu_{i}$ and $\sigma_{i}^{2}$ ) in rstan^9^ R-package (version 2.32.7) and run each of them with three Markov chains and 6000 iterations. The first half of the posterior samples was discarded (warm-up period), and then we made the inference with the remaining ones (9000 posterior samples). Convergence and efficiency were checked through Rhat and effective sample size.^24^ All models were run on a Dell laptop with 2.2 GHz Intel Core i7, 32 GB RAM, OS Windows.

**Figure 3.**
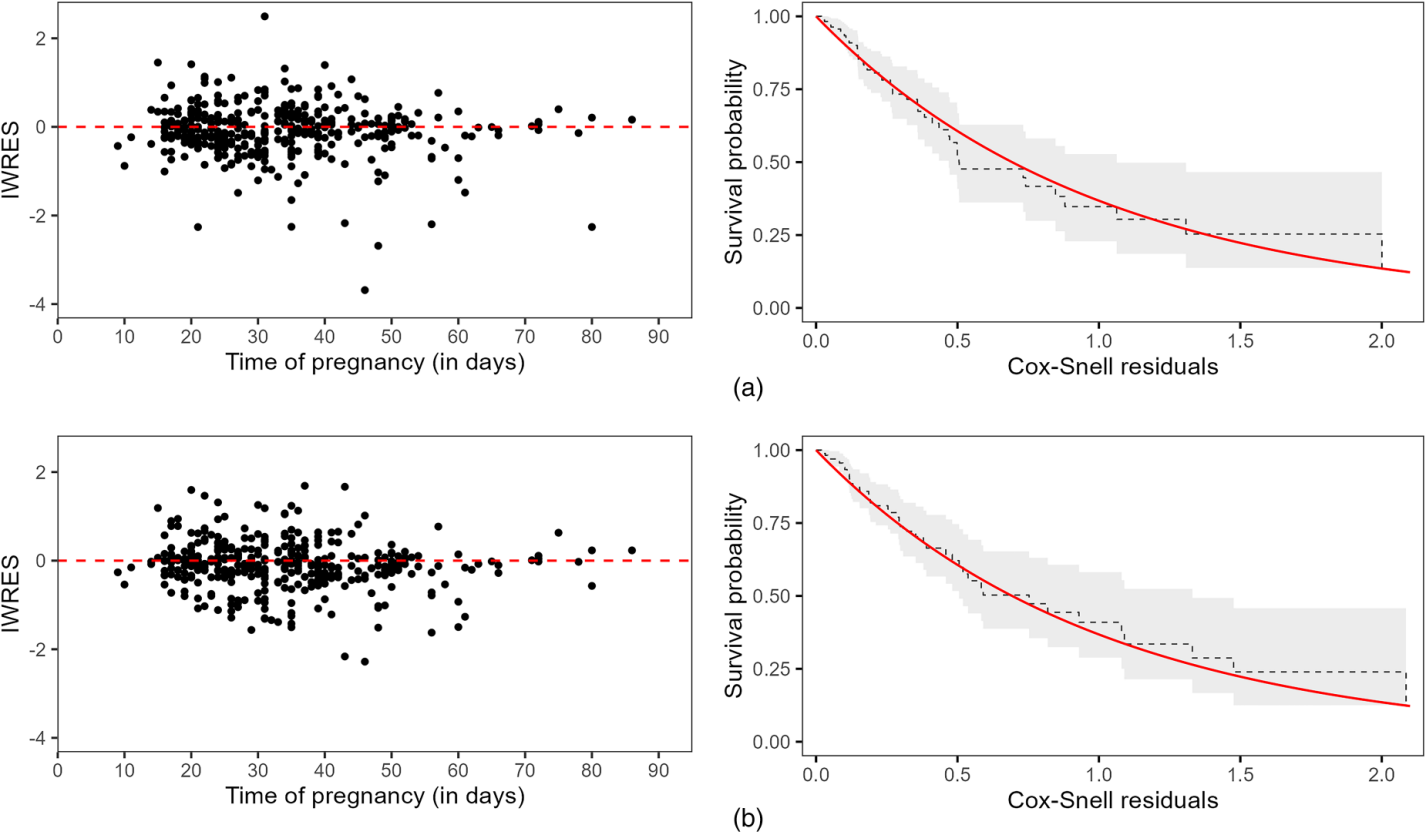
First column: Individual weighted residuals (IWRES). Second column: Kaplan–Meier estimates of the Cox–Snell residuals (dashed black line) and its 95% confidence interval (gray shadow), where the solid red line represents the survival function of the unit exponential distribution. (a) Longitudinal and survival residuals from joint model (1)-(2) (share $\mu_{i}$ ); (b) Longitudinal and survival residuals from joint model (4)-(5) (share $\mu_{i}$ and $\sigma_{i}^{2}$ ).

We analyzed the goodness-of-fit through longitudinal and survival residuals.^27^ Specifically, individual weighted residuals (IWRES) for longitudinal submodels and Cox-Snell residuals for survival submodels, considering interval-censored observations.^28^ Figure 3 shows both residuals by joint model.

In both cases, IWRES did not suggest any model misspecification, but it is possible to observe less dispersion of residuals considering the MELS specification (Figure 3b). Additionally, the Kaplan–Meier estimates of the Cox–Snell residuals were close to the theoretical survival curves (unit exponential distribution), indicating a suitable fit for both models.

We used the leave-one-out cross-validation (LOO-CV)^29^ to select the best joint model specification. This criterion is based on the out-of-sample prediction accuracy from a fitted Bayesian model using the log-likelihood evaluated at posterior simulations of the parameter values.^30^ Interpretatively, a lower LOO-CV value indicates a better model fit. It is worth noting that LOO-CV compares models based on their predictive performance, so it can be used for different classes of models, including non-nested specifications. Table 3 shows a posterior summary for both joint models as well as their respective LOO-CV.

**Table 3.** Posterior summary for the parameters of interest from joint models (1)-(2) (share $\mu_{i}$ ) and (4)-(5) (share $\mu_{i}$ and $\sigma_{i}^{2}$ ) using the three-parameter logistic specification (3).

Parameter	Joint model (1)-(2)		Joint model (4)-(5)	
	Mean	95% CI	Mean	95% CI
$a_{10}=\exp\;\{\theta_{1}\}$	4.620	(4.469, 4.788)	4.577	(4.446, 4.721)
$a_{20}=\exp\;\{\theta_{2}\}$	15.438	(14.139, 16.621)	15.712	(14.589, 16.726)
$a_{30}=\exp\;\{\theta_{3}\}$	7.196	(6.182, 8.348)	6.747	(5.927, 7.679)
$\alpha_{1}$	− 0.610	( − 1.027, − 0.190)	− 0.440	( − 0.839, − 0.029)
$\alpha_{2}$	–	–	3.149	(0.010, 8.212)
**LOO-CV**	612		392	
**Time (in minutes)**	27		80	

LOO-CV: leave-one-out cross-validation.

**Figure 4.**
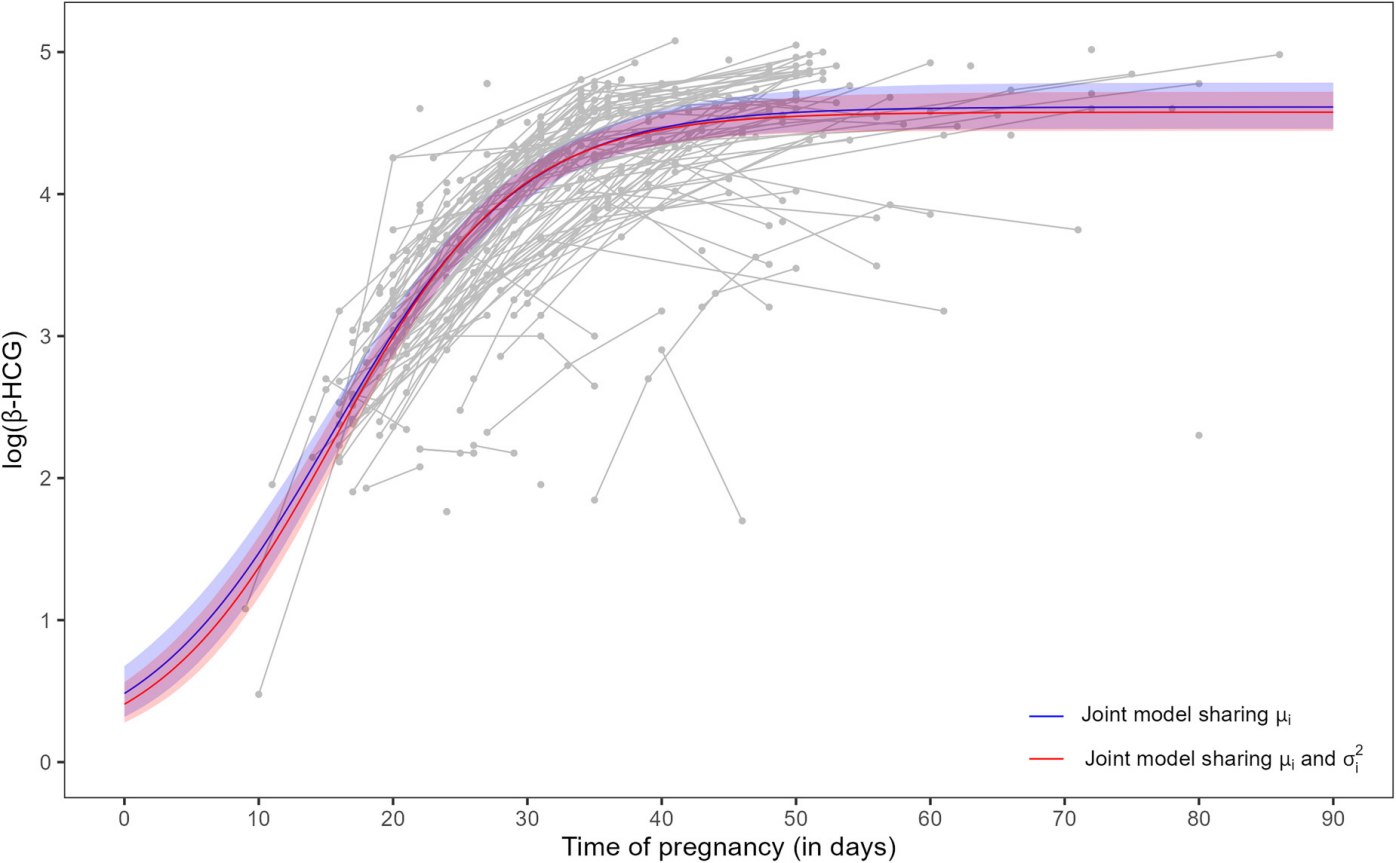
Posterior mean and 95% credible interval for the three-parameter logistic specification (3) from joint models (1)-(2) (share $\mu_{i}$ , blue color) and (4)-(5) (share $\mu_{i}$ and $\sigma_{i}^{2}$ , red color) using population parameters $a_{10}$ , $a_{20}$ , and $a_{30}$ (see Table 3). Trajectories in gray color are the longitudinal measurements of log( β -HCG) of the 173 women in the study (both groups). β -HCG: beta-human chorionic gonadotropin.

In Table 3, we can see that the population parameters, $a_{10}$ , $a_{20}$ and $a_{30}$ , that describe the median trajectory of the three-parameter logistic specification (3), are slightly different. Figure 4 shows such a trajectory using each joint model. In fact, the difference between the curves is minimal over the range of measurements.

Still in Table 3, the association parameter $\alpha_{1}$ , which connects the longitudinal process mean ( $\mu_{i}$ ) to the survival submodel, is negative for both joint models. This means that higher β -HCG hormone levels have a protective effect in terms of time until fetal death. This result corroborates the previous visual inspection (see Figure 1) that the abnormal group (women susceptible to miscarriage) presents longitudinal trajectories with lower β -HCG than the normal group. The joint model (4)-(5) also shows a strong and positive association ( $\alpha_{2}$ ) between the within-subject variance $\sigma_{i}^{2}$ and the time until fetal death. This is interpreted as greater intra-woman β -HCG variability leads to a higher risk of miscarriage. This result is also consistent with the observed pattern of β -HCG hormone levels in the abnormal group. In terms of model selection, the LOO-CV criterion indicates a better fit using the joint model that shares $\mu_{i}$ and $\sigma_{i}^{2}$ , but this model takes more than twice as long as the reference joint model.

## 6. Discussion

In this paper, we have proposed a Bayesian joint model based on a nonlinear MELS submodel for longitudinal data and a mixture cure submodel for interval-censored survival data. In addition, such submodels have shared terms described through the subject-specific longitudinal mean and variance.

We have compared our proposal with a reference joint model that shares only the subject-specific longitudinal mean. In the simulation study, our proposal has performed equivalently or better than the competing model. In the application, both approaches have shown suitable goodness-of-fit in terms of longitudinal and survival residuals (see Figure 3). However, the inclusion of within-subject variance as a shared term contributed to a better understanding of the β -HCG hormone pattern of women who had a miscarriage. Specifically, we have argued that increasing such variance leads to higher risks of fetal loss (see Table 3). Still, we highlight that this conclusion should be taken with extreme caution, as our study does not include baseline variables (not available) that could potentially be relevant risk factors.

Both joint models presented high computational times (27 and 80 minutes) given that our Chilean pregnancy miscarriage study has a relatively small sample size (173 women with few longitudinal measurements). These times may be reduced using two-stage strategies that preferentially correct the estimation bias.^31^

In conclusion, we hope this paper inspires other authors to consider all complex elements of real data in their joint modeling. In particular, we encourage researchers to adapt our codes for other problems, as well as to implement our joint model proposal in other statistical Bayesian model tools, such as JAGS^32^ and INLA.^33^
